# 1357. Initial and Peak Serum Levels of Krebs von den Lungen-6 for Prediction of Prognosis in Patients with COVID-19

**DOI:** 10.1093/ofid/ofad500.1194

**Published:** 2023-11-27

**Authors:** Geonui Kim, Hyeonwoo Kwon, Sang Hyun Ra, Eui Jin Chang, Seongman Bae, Jiwon Jung, Min Jae Kim, Yong Pil Chong, Sang-Oh Lee, Sang-Ho Choi, Yang Soo Kim, Sung-Han Kim

**Affiliations:** Asan Medical Center, Songpa-gu, Seoul-t'ukpyolsi, Republic of Korea; Asan Medical Center, Songpa-gu, Seoul-t'ukpyolsi, Republic of Korea; Asan Medical Center, Songpa-gu, Seoul-t'ukpyolsi, Republic of Korea; Department of Internal Medicine, Asan Medical Center, Seoul, Korea, Seoul, Seoul-t'ukpyolsi, Republic of Korea; Asan Meidical Center, Songpa-gu, Seoul-t'ukpyolsi, Republic of Korea; Asan Medical Center, Songpa-gu, Seoul-t'ukpyolsi, Republic of Korea; Asan Medical Center, Songpa-gu, Seoul-t'ukpyolsi, Republic of Korea; Asan Medical Center, Songpa-gu, Seoul-t'ukpyolsi, Republic of Korea; Asan Medical Center, Songpa-gu, Seoul-t'ukpyolsi, Republic of Korea; Asan Medical Center, Songpa-gu, Seoul-t'ukpyolsi, Republic of Korea; Asan Medical Center, Songpa-gu, Seoul-t'ukpyolsi, Republic of Korea; Asan medical center, Seoul, Seoul-t'ukpyolsi, Republic of Korea

## Abstract

**Background:**

Krebs von den Lungen-6 (KL-6) has been reported to be associated with prognosis in patients with COVID-19. However, there is limited data on the correlation between COVID-19 prognosis and varying KL-6 levels at different time points. We investigated the optimal cutoff values of both initial and peak serum KL-6 levels in order to predict mortality, and also examined their correlation with mortality within 30 days.

**Methods:**

In this retrospective cohort study, data on serially collected serum KL-6 levels in patients hospitalized with COVID-19 between December 2020 and January 2022 at a single tertiary hospital in South Korea were collected. The area under the ROC curve and Youden index were used to determine the cutoff points for the initial and peak KL-6 levels that best predicted 30-day mortality. The association between both initial and peak KL-6 values with 30-day mortality were assessed by univariate and multivariate logistic regression model.

**Figure:**
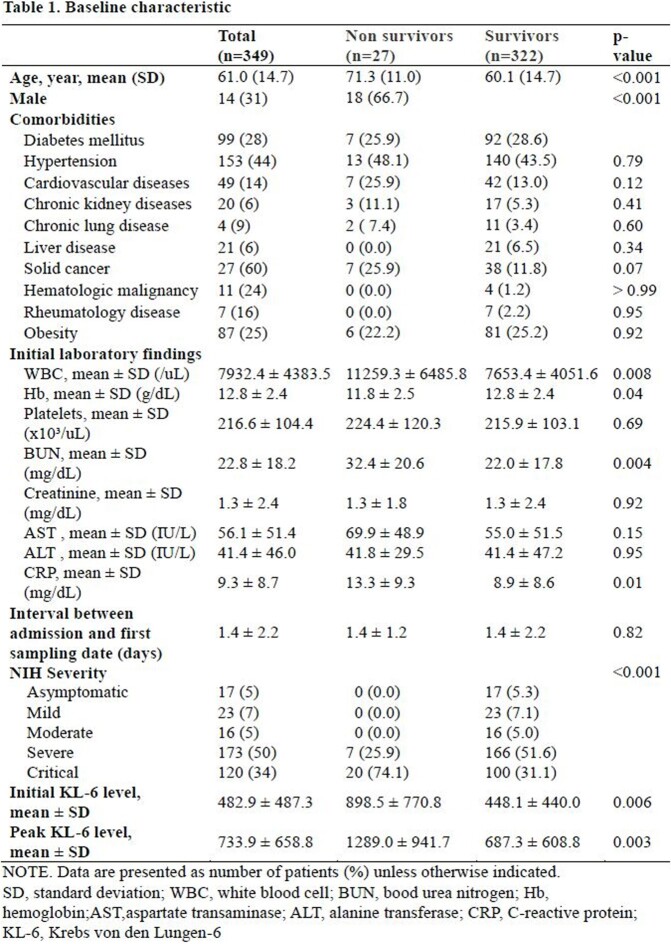

**Results:**

A total of 349 patients were included, and among them, 27 individuals died within 30 days, resulting in a mortality rate of 7.7%. The mean initial and peak KL-6 levels were significantly higher in the non-survivor group compared to the survivor group (898.5 vs. 448.1, p=0.006; 1289.0 vs. 687.3, p=0.003, respectively). The initial and peak KL-6 values that best predicted 30-day mortality were 491.85 U/mL (sensitivity 72.7%, specificity 74.1%, area under curve [AUC] 0.748) and 660.05 U/mL (sensitivity 63.0%, specificity 81.5%, AUC 0.744), respectively. Initial KL-6 greater than 491.85 U/mL (odds ratio [OR], 7.60; 95% CI, 3.10 to 18.59) and a peak KL-6 greater than 660.05 U/mL (OR 7.55; 95% CI 2.56 to 22.26) were found to be significantly associated with 30-day mortality, independent of age, solid cancer, and elevated serum C-reactive protein ( > 7.5 mg/dL).

**Figure:**
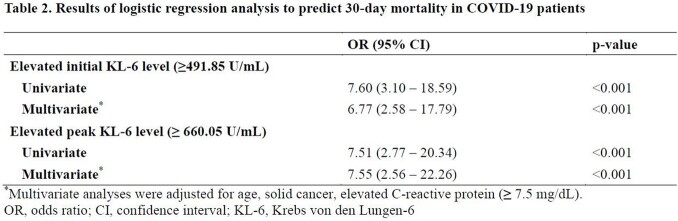

**Figure:**
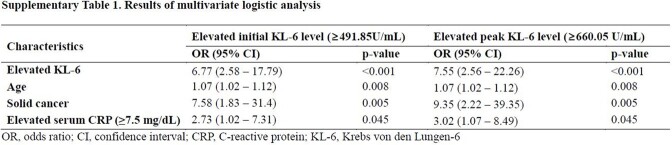

**Figure:**
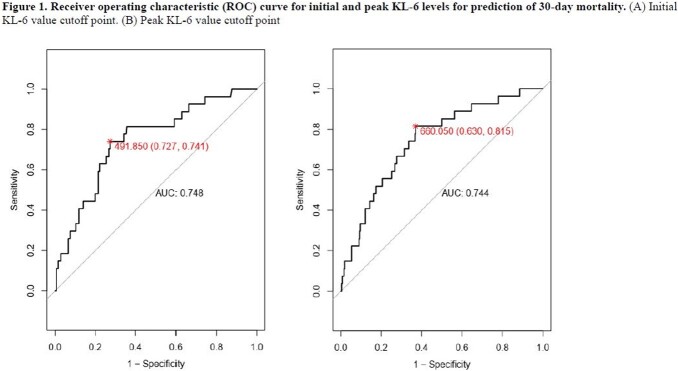

**Conclusion:**

We found that both initial and peak levels of KL-6 were significantly related to 30-day mortality in hospitalized COVID-19 patients. These findings suggest that serially monitoring blood KL-6 levels could serve as a valuable prognostic indicator for COVID-19.

**Disclosures:**

**All Authors**: No reported disclosures

